# Case report: Positioning head tilt observed in a dog and four cats with bilateral peripheral vestibular dysfunction

**DOI:** 10.3389/fvets.2024.1495807

**Published:** 2024-11-18

**Authors:** Shinji Tamura, Yuya Nakamoto, Koen M. Santifort, Yumiko Tamura

**Affiliations:** ^1^Tamura Animal Clinic, Hiroshima, Japan; ^2^Neuro Vets Animal Neurology Clinic, Kyoto, Japan; ^3^IVC Evidensia Referral Hospital Arnhem, Arnhem, Netherlands; ^4^IVC Evidensia Referral Hospital Hart van Brabant, Waalwijk, Netherlands

**Keywords:** positioning head tilt, dog, cat, bilateral vestibular, peripheral, otitis interna, idiopathic vestibular disease

## Abstract

Positioning head tilt (PHT) is a dynamic neurologic sign that occurs when the head tilts in the opposite side of a voluntary lateral turn of the head. Notably, a head tilt is absent when the head is held stationary or when the animal is moving forward. PHT is thought to be caused by a lack of inhibitory input to the vestibular nuclei due to dysfunction of the cerebellar nodulus and uvula (NU). NU dysfunction is proposed to not only be caused by pathologies that affect the NU itself, but also by reduced input of proprioceptive information from the spindles of cervical muscles. As an example of the former, it has been noted in dogs with hypoplasia of the cerebellar nodulus and uvula (NU), dogs with lysosomal storage diseases, and in a dog with a cerebellar tumor. As an example of the latter, it has been observed in feline cases of hypokalemic myopathy and myasthenia gravis. In this study, we describe and discuss our observations of PHT in one dog and four cats with lesions affecting the peripheral vestibular apparatus bilaterally.

## 1 Introduction

Positioning head tilt (PHT) in veterinary neurology is a clinical sign that occurs when the head tilts in the opposite side of a voluntary lateral turn of the head (e.g., tilting to the left when the head moves to the right and vice versa) (1, 2). Notably, a head tilt is absent when the head is held stationary or when the animal is moving forward. PHT has been documented in various cases, including three dogs with cerebellar hypoplasia [specifically of the nodulus and uvula (NU)] (1), a dog with a tumor invading the NU (3–5), and nine dogs with lysosomal disease (6). In the latter, the entire brain including the NU was atrophic. The PHT is thought to be caused by a lack of inhibitory input to the vestibular nuclei due to NU dysfunction. Additionally, PHT has been reported in 14 cats with hypokalemic myopathy (7) and 2 cats with myasthenia gravis (8). In these cases, the mechanism of PHT is believed to involve altered function of muscle spindles in the rectus and obliquus capitis muscles. This alteration possibly leads to disrupted proprioceptive information from the spindles to the NU, hindering the NU's ability to inhibit the excitation of rostral, medial, and caudal vestibular nuclei induced by head movement (7, 8).

Bilateral peripheral vestibular diseases occur occasionally in dogs and more frequently in cats, often resulting from bilateral otitis interna or idiopathic vestibular disease (9). Affected animals typically crouch low to the ground, walk tentatively, and may fall to both sides (9). Unlike unilateral vestibular conditions, these animals do not exhibit head tilt or pathological nystagmus and lack vestibulo-ocular reflexes. Instead, they often display wide lateral head excursions, interpreted as attempts to stabilize the visual field in the absence of physiological nystagmus (9).

In this study, we describe the cases of one dog and four cats with bilateral peripheral vestibular diseases presenting with PHT. The mechanism of PHT in these animals is discussed.

## 2 Case description

### 2.1 Case 1

**Figure 1:**
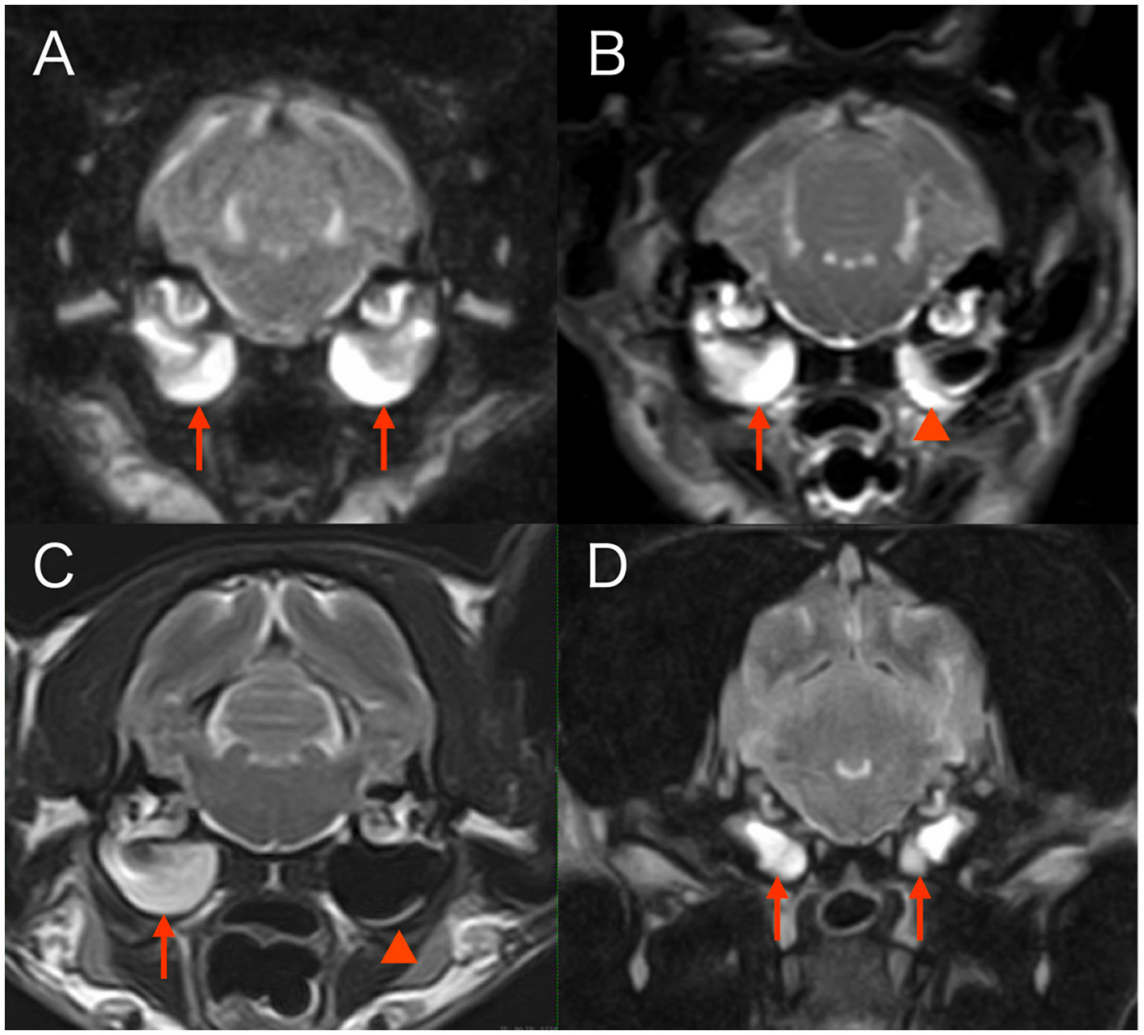
Transverse T2-weighted magnetic resonance images at the level of tympanic bullae. **(A)** Case 1. Both tympanic cavities are almost completely filled with fairly homogenous material hyperintense to gray matter (arrows). **(B)** Case 3. The right tympanic cavity is almost completely filled with fairly homogenous material hyperintense to gray matter (arrow). Material of similar signal intensity adheres along the walls of the left tympanic cavity (arrowhead). **(C)** Case 4. The right tympanic cavity is completely filled with fairly homogenous material hyperintense to gray matter (arrow). A faint crescent of material of similar signal intensity is present along the ventral border of the left tympanic cavity (arrowhead). **(D)** Case 5. Both tympanic cavities are completely filled with fairly homogenous material hyperintense to gray matter (arrows).

A 14-year-and-8-month-old female neutered domestic short-haired (DSH) cat was referred to Tamura Animal Clinic for detailed examination of ataxia and wide lateral head excursions which had been present for an unknown period of time. The cat had been treated with steroids (details unknown) for unilateral head tilt (side unknown) during the previous 2 months at another hospital. When wide lateral head excursions were observed, PHT was observed concurrently (Supplementary Video 1). Neurological examination revealed vestibular ataxia, loss of vestibulo-ocular reflexes, and unresponsiveness to sound. The neuroanatomical lesion localization was: peripheral vestibulocochlear apparatus, bilaterally. The complete blood count (CBC) revealed no abnormalities. Serum biochemistry revealed increased alanine transaminase activity [132 IU/L; reference interval (RI) 18–51 IU/L]. MRI findings were consistent with bilateral otitis media/interna (0.3T Hitachi Airis II comfort) (9–11) (Figure 1). Cerebrospinal fluid (CSF) was not collected due to financial constraints. Medical treatment with cefovecin sodium (8 mg/kg s.c., once every 2 weeks) and marbofloxacin (8.5 mg/kg, p.o., sid) was prescribed for 10 weeks, but the signs did not change at the end of treatment.

### 2.2 Case 2

An 8-year-old female neutered DSH cat was referred to Tamura Animal Clinic with signs of horizontal nystagmus with fast phase to the right and lateral head excursions that had been observed for 10 days before referral. The cat had a subcutaneous ureteral bypass system which had been implanted for treatment of ureteral calculus 3 years earlier by the referring veterinarians. Additionally, polyarthritis had been diagnosed 4 months prior and prednisolone was administered since (dose at time of referral: 1.5 mg/kg once daily). Two and a half months before presentation, the cat had started showing signs consistent with a unilateral vestibular disorder. Specifically, signs reported in medical files and by the owner included a spontaneous pathological nystagmus, head tilt, wide-based stance, and vestibular ataxia. Details on the side of the abnormalities were not noted in the files. Thereafter, the vestibular signs had gradually improved. When the current signs were noticed, 10 days before referral, the prednisolone was reduced to 0.8 mg/kg once daily. At the time of arrival at Tamura Animal Clinic, although a detailed neurological examination could not be performed due to the aggressive nature of the cat, neither pathological nor physiological nystagmus was observed. PHT was observed during head excursions (Supplementary Video 1). The neuroanatomical lesion localization was: peripheral vestibular apparatus, bilaterally. CBC and serum biochemistry revealed no abnormalities. MRI revealed no abnormalities in the brain, middle ear, or inner ear (0.3T Hitachi Airis II comfort). CSF was not collected due to financial constraints. A diagnosis of bilateral idiopathic vestibular disease was made. No specific treatment was given. Vestibular ataxia improved, but there was no change in head movement. Four months later, the cat died as a consequence of progressive anemia of unknown cause.

### 2.3 Case 3

A 12-year-and-5-month-old female neutered DSH cat presented with a right-sided head tilt and horizontal nystagmus with fast phase to the right that had been present for the last 3 months. The patient was treated with steroids (details unknown) at another hospital. Two months after onset, the head tilt was less noticeable but persistent. The cat was referred to Neuro Vets Animal Neurology Clinic with signs of vestibular ataxia most prominently noticeable in the pelvic limbs and wide lateral excursions of the head that had been noticed for 2 weeks before. When the cat walked, mild PHT was observed (Supplementary Video 2). Neurological examination revealed bilateral, but slightly asymmetrical vestibular ataxia (tendency of leaning to the left with the pelvic limbs and caudal trunk), loss of vestibulo-ocular reflexes, and unresponsiveness to sound. The neuroanatomical lesion localization was: peripheral vestibulocochlear apparatus, bilaterally. CBC and serum biochemistry revealed no abnormalities. MRI findings were consistent with bilateral otitis media/interna (0.4T Hitachi APERT Lucent) (9–11) (Figure 1). CSF was not collected due to financial constraints. Medical treatment with amoxicillin clavulanate (30 mg/kg, p.o., bid) was initiated for 8 weeks. The neurologic signs did not resolve but were less severe over the course of treatment and after treatment at the 8-week follow-up.

### 2.4 Case 4

A 15-year-old female neutered DSH cat was presumptively diagnosed with bilateral otitis media/interna 1 week prior, directly after topical medication (Neptra, Elanco) had been applied in both ears for a diagnosis of otitis externa (based on otoscopic findings) bilaterally at another hospital. Tympanic membrane integrity or rupture was not reported in the medical files. Directly after topical medical treatment, cat showed severe bilaterally symmetrical vestibular ataxia, drooling, and bilateral Horner syndrome (miosis, enophthalmos, ptosis, and protrusion of the third eyelid), and had become unresponsive to sound. Directly afterwards, the ears were flushed and prednisolone (1 mg/kg, p.o., sid) and metoclopramide (0.2 mg/kg, p.o., tid) were administered by the referring veterinarian. The ataxia gradually improved but other signs persisted and the cat was referred to the neurology department of IVC Evidensia Referral Hospital Arnhem. Neurological examination revealed bilaterally symmetrical vestibular ataxia, wide lateral head excursions, PHT (Supplementary Video 1), bilaterally mildly decreased palpebral reflexes, bilateral Horner syndrome, loss of vestibulo-ocular reflexes, and unresponsiveness to sound. The neuroanatomical lesion localization was: peripheral vestibulocochlear apparatus, bilaterally. CBC and serum biochemistry revealed no abnormalities. MRI (1.5T Canon Vantage Elan), otoscopy findings, and clinical history were consistent with a diagnosis of bilateral tympanic membrane disruption, ototoxicity, and (suspectedly sterile) otitis media/interna (Figure 1) (9–11). CSF was not collected. After flushing of the tympanic cavities, the patient was treated medically with topical auricular medication every other day (honey ear drops, Dermiel^®^, AST Farma B.V., The Netherlands, and triamcinolone acetonide 0,1%), topical ear cleaner once every 2 weeks (VetSoothe Clear^®^), and oral dexamethasone (0.08 mg/kg, p.o., sid) for 1 week. Topical auricular medications were tapered and stopped after 2 months. At 4-month follow-up, persistent bilaterally symmetrical vestibular ataxia was still noticed and the cat remained unresponsive to sound. Signs of Horner syndrome were less apparent (mild ptosis, miosis, and protrusion of the third eyelid were still present).

### 2.5 Case 5

A 3-year-and-8-month-old female French bulldog was referred to Tamura Animal Clinic for sudden onset of vomiting, rotatory nystagmus, and abnormal head and trunk movements that had started 2 days before referral. The dog had difficulty standing up, and repeatedly and rapidly rolled the head from side to side with the abdomen on the floor. The trunk also rolled from side to side (Supplementary Video 3). Neurological examination revealed severe bilaterally symmetrical vestibular ataxia, head shaking, loss of vestibulo-ocular reflexes, and unresponsiveness to sound. The neuroanatomical lesion localization was: peripheral vestibulocochlear apparatus, bilaterally. CBC and serum biochemistry revealed elevated C-reactive protein (CRP) concentration (>7.0 mg/dL; RI <0.3). MRI findings were consistent with bilateral otitis media/interna (Figure 1) (9–11), inflammatory changes around the bulla tympanica on the right, and focal meningitis in the area of the right brain stem (0.3T Hitachi Airis II comfort). CSF was not collected due to financial constraints. Medical treatment with maropitant citrate monohydrate (1 mg/kg, s.c. sid), cefovecin sodium (8 mg/kg s.c.), orbifloxacin (5 mg/kg s.c. sid), and subcutaneous fluid (for 6 days) until the dog had a satisfactory oral fluid and food intake. One week after the start of treatment, the patient was able to walk, but head excursions and PHT were observed (Video 3). CRP concentration had dropped to within RI. Additional cefovecin sodium (8 mg/kg s.c. once every 2 weeks) and marbofloxacin (6.9 mg/kg, p.o., sid) were administered for 8 weeks. Eight weeks later, residual neurological deficits were present, consisting of mild bilaterally symmetrical vestibular ataxia, head excursions, and PHT. At reevaluation 4 years later, mild bilaterally symmetrical vestibular ataxia remained, but head excursions and PHT had disappeared. It is unclear when the PHT had disappeared.

## 3 Discussion

The neural pathway for the maintenance of head equilibrium is described as follows. In a resting animal, the left and right otoliths receive symmetrical gravitational stimulation. This stimulation is transmitted through the vestibular nerve to the left and right lateral vestibular nuclei, which maintain head equilibrium by equally contracting the oblique and rectus capitis muscles, the antigravity muscles of the head, on both sides through the ipsilateral lateral vestibular spinal tract. When the head moves to the left, the rostral, medial, and caudal vestibular nuclei detect the shift in the center of gravity to the left, mainly through the input from the left and right semicircular canals. To maintain equilibrium, the left side of the body through the medial vestibulospinal tract increases the tone of the left-sided antigravity muscle groups and the right side decreases it, thereby preventing the body from falling to the left (12). Additionally, the responses of vestibular neurons are influenced by the current behavior and motor function of the animal. Vestibular neuron activity is modulated not only by input from the peripheral vestibular apparatus but also by proprioceptive receptive afferents and efferent signals from the motor nuclei and cortex during active movements (13).

When the head moves laterally to the left, the NU compares the input from the vestibular apparatus through the vestibulocerebellar tract with the input from the muscle spindles of the neck muscles through the spino-cuneocerebellar tract. This comparison provides relative positional information between the head and the trunk. The NU then outputs signals to inhibit the overexcitation of the left-sided reflexive rostral, medial, and caudal vestibular nuclei. Dysfunction of the NU can cause a head tilt to the right due to an excessively high tension of the left-sided antigravity muscles (1). This phenomenon highlights the importance of the NU receiving input from both the vestibular apparatus and the muscle spindles of the cervical muscles for its inhibitory function (12). In conditions like hypokalemic myopathy and myasthenia gravis in cats, reduced input from the cervical muscle spindles due to muscle spindle dysfunction can result in PHT (7, 8). In the five cases presented here, none of the patients were considered to have a dysfunction of NU or a muscular disease. In cases with bilateral peripheral vestibular dysfunction, head movement is detected by cervical muscle spindles. Stimulation of the medial vestibulospinal tract increases the tension of the oblique and rectus capitis muscles on the side toward which the head turns (1). However, the NU does not produce an inhibitory output because the input from the vestibular apparatus to the NU is lost, resulting in the PHT (7, 8). Interestingly, PHT in Case 5 disappeared at reevaluation 4 years later. Similarly, in a canine case of cerebellar hypoplasia, PHT also resolved, which was postulated to be due to “compensation” (1). Bilateral vestibular signs are known to be compensated over time (14, 15), suggesting that some form of cerebello-vestibular system compensation might explain the disappearance of the PHT in Case 5.

We postulate that in our five reported cases, it was the lack of input from the peripheral components of the vestibular system (i.e., the inner ear structures and vestibulocochlear nerve) that lead to NU dysfunction and the clinical signs of PHT. For two of the five patients (cases 2 and 5), we consider it unlikely that both peripheral vestibular systems were affected at the same time. We propose that the unilateral vestibular involvement first resulted in unilateral peripheral vestibular signs and then the other, previously normally functioning vestibular system was affected culminating in bilateral peripheral vestibular dysfunction. These cases showed pathological nystagmus at the second occasion. We hypothesize that some degree of compensation had occurred after the initial onset of unilateral vestibular dysfunction. When the unilateral peripheral vestibular system is damaged, input from that side of the peripheral vestibular system is absent. Signal transmission to the vestibular nuclei neurons on the damaged side is markedly reduced to absent. Vestibular compensation is thought to occur due to the release of inhibition through commissural fibers from the vestibular nuclei of the healthy side to the affected side, resulting in an increase in spontaneous firing, and also input of rotational information from the semicircular canal of the healthy side (16). Up to the time of loss of peripheral vestibular input from the previously healthy side, bilateral vestibular nuclei were active once again (the function of the nuclei on the affected side “compensated”). Loss of peripheral vestibular input from the previously healthy side then results in a new imbalance of activity in both vestibular nuclei and pathological, spontaneous nystagmus. The eventual disappearance of pathological nystagmus afterwards could then be explained by another “round” of compensation.

The repeated and rapid rolling of the head and trunk to the left and right with the abdomen on the floor observed on the second day in Case 5 has not been previously reported. However, similar signs have been described in laboratory cats within 0–2 days following bilateral labyrinthectomy (14, 15). In these studies, cats were able to sit up 1–2 days post-operation, and head oscillations, referred to as “excursions” in the literature, were noted (14, 15). These head oscillations included a slight rolling component, although the side was not described. The PHT observed in the present study may correspond to the rolling component these authors referred to (14, 15). The period from the 2^nd^ to 7^th^ day post-operation was marked by rapid recovery, with standing and walking abilities being restored. Weeks 2–6 represented the slow recovery phase, during which most functions returned, aside from persistent mild ataxia. Four months after the operation, oscillations of the head were no longer apparent in those experimentally lesioned cats (14, 15).

This study has some limitations, including the small number of cases. A more detailed observation of clinical signs and progression in a larger cohort with similar characteristics is warranted. Another limitation is that the diagnosis and treatments of bilateral otitis interna in three cats and one dog was based solely on the history, clinical findings, and MRI findings. Culture and sensitivity testing of middle ear content or flushes was not performed in those cases. Although a diagnosis of infectious otitis interna is not formally confirmed (which would require either rigorous sampling or histopathology), the presence of clinical signs such as loss of physiological nystagmus and unresponsiveness to sound supported the diagnosis of bilateral vestibulocochlear dysfunction. Together with the MRI findings, the diagnoses of otitis media/interna in this report align with those in other reports and that are accepted within the veterinary neurology community (9, 10, 17, 18). Antibiotic treatment for otitis media/interna should, when possibly, be based on sensitivity testing (17). However, unfortunately, even when cultures are performed they are often negative even though a bacterial cause is still highly suspected (9, 11, 17, 18). Empirical choices, clinician preferences, and generally broad-spectrum antibiotics therefore come into play when treating cases of (suspected) bacterial otitis media/interna.

In conclusion, this report identified a third lesion site (bilateral peripheral vestibular apparatus) capable of producing PHT, in addition to the previously reported NU and bilateral cervical muscle spindles. Although hypothetical, all of these lesion sites are part of the system that maintains head equilibrium during head movement and any lesion affecting these structures might result in PHT. These hypotheses may be strengthened through further observation of clinical cases.

## Data Availability

The original contributions presented in the study are included in the article/supplementary material, further inquiries can be directed to the corresponding author.

## Supplementary material

Supplementary Video 1 Cases 1, 2, and 4-Positioning head tilt (the head tilts in the opposite side of a voluntary lateral turn of the head).

Supplementary Video 2 Case 3-Mild positioning head tilt (the head tilts in the opposite side of a voluntary lateral turn of the head).

Supplementary Video 3 Case 5-Head tilting from side to side and trunk twisting from side to side, and positioning head tilt (head tilts in the opposite side of a voluntary lateral turn of the head).
